# Erratum to “A watershed model of individual differences in fluid intelligence” [Neuropsychologia 91 (2016) 186–198]

**DOI:** 10.1016/j.neuropsychologia.2017.09.032

**Published:** 2017-11

**Authors:** Rogier A. Kievit, Simon W. Davis, John Griffiths, Marta M. Correia, Cam-CAN, Richard N. Henson

**Affiliations:** a MRC Cognition and Brain Sciences Unit, 15 Chaucer Rd, Cambridge CB27EF, United Kingdom; b Department of Psychology, University of Cambridge, Downing Street, Cambridge CB23EB, United Kingdom; c Center for Cognitive Neuroscience, Duke University, Durham, NC 27708, United States; d Rotman Research Institute, Baycrest, Toronto, Ontario, Canada M6A2E1; e Cambridge Centre for Ageing and Neuroscience (Cam-CAN), University of Cambridge and MRC Cognition and Brain Sciences Unit, Cambridge, United Kingdom

The publisher regrets that due to an error the full text of Appendix A was missing in the original publication. The missing text is included below.

The publisher would like to apologise for any inconvenience caused.

## Appendix A

Estimating WM ROIs

Diffusion-weighted images (DWI) were acquired at the MRC Cognition and Brain Sciences Unit, using a 3T Siemens TIM Trio MRI scanner, and a 32-channel head coil. A twice-refocused spin-echo sequence was used to minimise eddy-currents, with 30 uniformly spaced gradient directions for each of two b-values (1000 and 2000 s/mm^2^), and three non-diffusion weighted images (b-value = 0). Other imaging parameters were: TR = 9100 ms, TE = 104 ms, 2 × 2 × 2 mm^3^ resolution, FoV 192 × 192 mm^2^, 66 axial slices and GRAPPA acceleration factor of 2. A structural MPRAGE was also acquired for each participant (see Shafto et al. (2014) for sequence details). Traditionally, DWI data is motion corrected at the postacquisition level by using image registration techniques to co-register each diffusion-weighted image to the first acquired b = 0 image. However, as discussed in Ben-Amitay et al. (2012), when high b-values are used, this technique will fail to correct for distortions and motion, and may introduce other artefacts. For this reason, we did not apply registration-based motion correction to the DWI data in this study. Detection and exclusion of outliers was performed at the analysis level to avoid including datasets affected by other artefacts and distortions. All pre-processing and modelling of MRI data was performed using a combination of functions from FSL version 5.0.8 (Jenkinson et al., 2012), SPM12 (http://www.fil.ion.ucl.ac.uk/spm/software/spm12/), and custom scripts written in C and Matlab, integrated in the Automatic Analysis (aa) package (Cusack et al., 2014). After removal of non-brain tissue, a non-linear diffusion tensor model was applied to the DWI data. Non-linear fitting of the diffusion tensor provides better noise modelling than standard linear model fitting, resulting in more accurate and un-biased estimates of the diffusion tensor and its different metrics (Jones and Basser, 2004). The diffusion tensor’s eigensystem was used to compute the fractional anisotropy (FA) at each voxel. The FA maps were then spatially normalised into a standard stereotactic space as follows: firstly, the average of the three b = 0 images for each subject was coregistered to their MPRAGE; secondly, the MPRAGE images were coregistered across participants to a sample-specific template using DARTEL (Ashburner, 2007), and the transformations so derived were used to warp each participant’s images into standard MNI space, including their FA maps. The resulting FA images in MNI space were smoothed with a 1 mm FWHM Gaussian kernel to reduce residual interpolation errors.
