# Supplementary figures and images for: A watershed model of individual differences in fluid intelligence
Source: Neuropsychologia. 2016 Oct;91:186–98. doi: 10.1016/j.neuropsychologia.2016.08.008 (PMC5081064; doi:10.1016/j.neuropsychologia.2016.08.008)

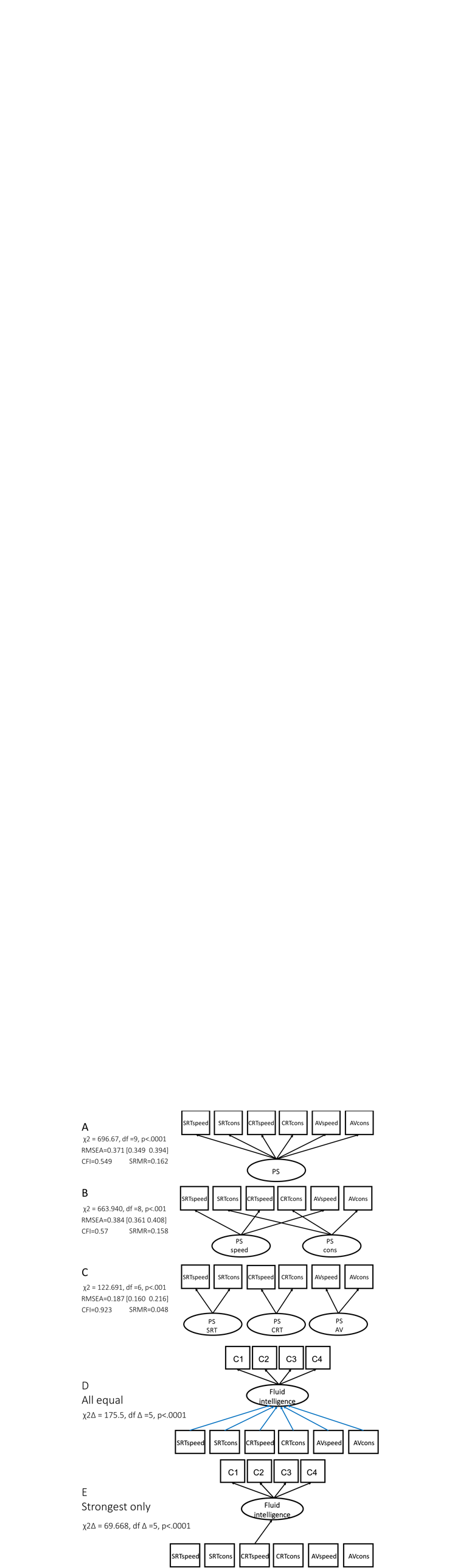

Supplement: Supplementary file 3 — Supplementary material [file mmc3.zip › mmc3.tif]

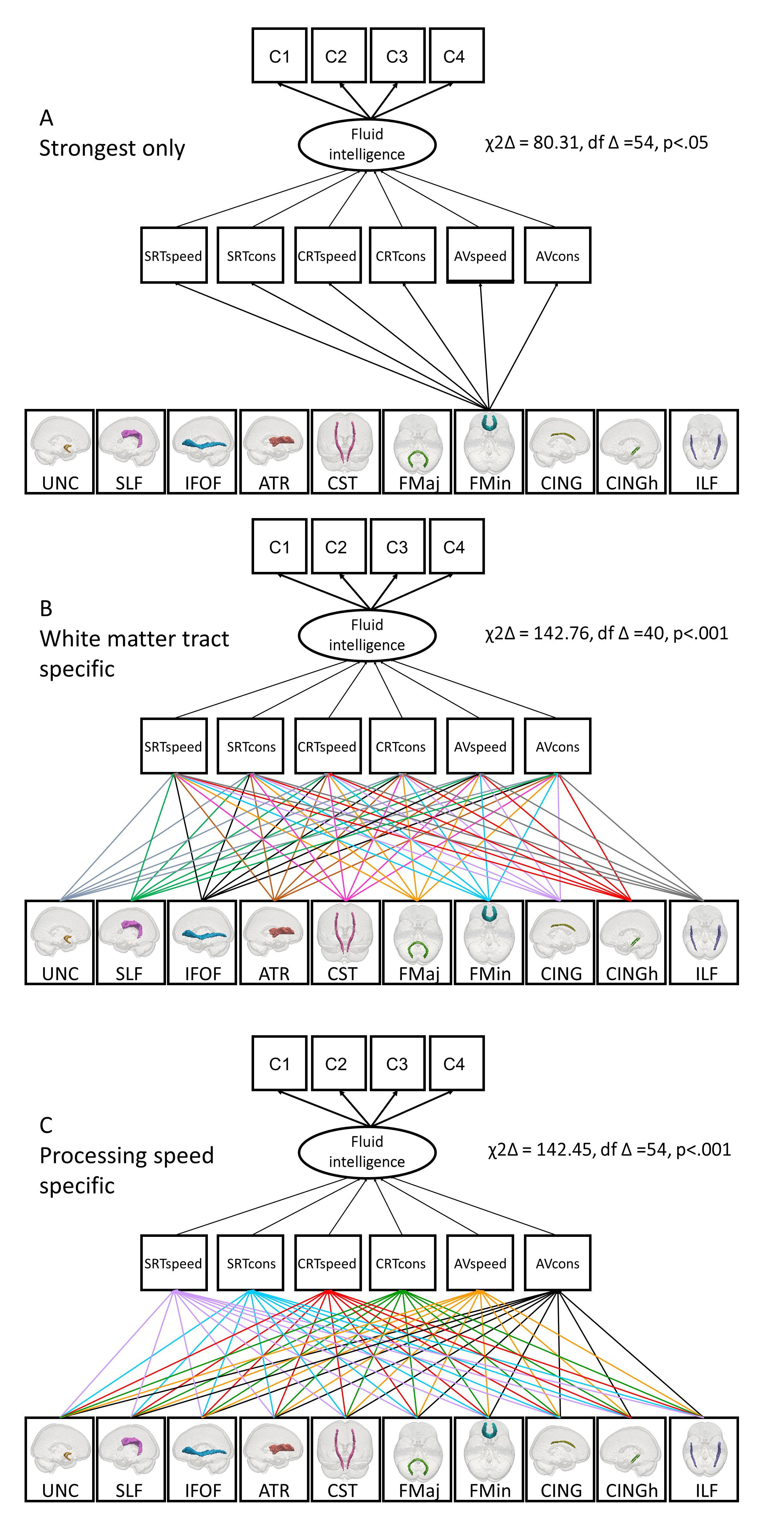

Supplement: Supplementary file 4 — Supplementary material [file mmc4.zip › mmc4.tif]
